# Biomechanics of Topspin Forehand Loop in Table Tennis: An Application of OpenSim Musculoskeletal Modelling

**DOI:** 10.3390/healthcare11091216

**Published:** 2023-04-25

**Authors:** Ruizhe Zhu, Xiaoyi Yang, Luis C. Chong, Shirui Shao, Bíró István, Yaodong Gu

**Affiliations:** 1 Faculty of Sports Science, Ningbo University, Ningbo 315211, China; 2 Faculty of Engineering, University of Szeged, 6720 Szeged, Hungary

**Keywords:** table tennis, forehand loop, lower extremity, different levels, OpenSim

## Abstract

Topspin is one of the most attacking strokes in table tennis, and topspin forehand loop is an effective way to score. The aim of this study was to investigate the kinematics of the lower extremities in topspin forehand loop between different levels via OpenSim Musculoskeletal Modelling. Ten elite athletes (NL1) and ten medium athletes (NL2) performed the topspin forehand loop without muscle and joint injuries. An eight-camera Vicon motion capture system was used to measure the kinematics data. During the topspin forehand loop, the forward phase (FP) and the entire phase (EP) of the NL1 were significantly shorter than that of the NL2. In the sagittal plane, NL1 significantly had greater hip and ankle flexion and extension at range of motion (ROM) but less hip flexion and knee flexion at FP and less ankle flexion at BP than NL2. In the frontal plane, NL1 displayed less ROM in the hip joint and significantly less hip abduction ROM at the backward phase (BP). In the transverse plane, NL1 had a significantly greater ROM in the hip joint and displayed significantly less hip ROM at the BP. The level differences presented in this study could help table tennis athletes to improve performance and coaches to develop technical training.

## 1. Introduction

Table tennis is a racket sport [1,2] in which technology is considered to be an important performance factor [3]. There are many technical skills, such as strokes. Types of table tennis strokes include the forehand, backhand, smash, push, and so on [4]. During competition, a high-level table tennis player hits the ball in a short amount of time and has enough time to hit the next ball [5]. In recent years, table tennis technology has been constantly advancing [6], such as the diameter of table tennis ball changing from 38 mm to 40+ mm. It is difficult for professional table tennis athletes to score directly in one round of matches; thus, high-level table tennis players in training and competition should pay a great deal of attention to the practice of this technique to continuously improve the quality and stability and strengthen the ability to apply it in the actual combat of the game [7,8]. Therefore, table tennis athletes need to improve the quality of their strokes to create favorable conditions and to pose a greater threat to their opponents in table tennis matches. 

The topspin forehand loop is known as one of the most aggressive ways [3] because of its strength, fast speed, and strong rotation [9]. The topspin forehand loop has become essential for table tennis athletes [10], and the characteristics of the topspin forehand loop made it become the most common and frequently used attacking technique in table tennis [11]; if it is well practiced in daily training, it performs well in formal matches [12,13]. The biomechanics in table tennis stroke techniques have been studied extensively. Li et al. found that the energy cost of the forehand loop drive decreases at higher stroke frequencies. The high repetition forehand loop drive task was demonstrated to be aerobically dominant with the anaerobic energy system playing a vital role, and the anaerobic lactic system has limited importance [14]. Iino and Kojima found the internal rotation torque at the shoulders joint and pelvis has an impact on the performance of balls [15]. Seemiller et al. found that the importance of small muscles around the shoulders, elbow, and wrist joints in performing the forehand loop stroke [16]. Malagoli Lanzoni compared the upper limbs biomechanical characteristics of the table tennis topspin when competitive table tennis players played cross court (CC) and long line (LL). The research showed that kinematic differences exist between the LL and the CC topspin forehand in table tennis athletes [17].

There has been much research on the forehand loop, not only on the upper limb but also on the lower limb. Malagoli Lanzoni found differences in the footwork of table tennis players from different regions. Asian players used footwork more frequently than European players both during training and in competition [12]. Yang et al. found that males had greater hip and knee flexion angles during the entire motion phase and greater internal rotation angles of the hip during the forward swing phase. The joint stiffness of knees in males was greater than females in the frontal plane. Females in the forward swing phase showed greater hip flexion, adduction, and internal rotation moments than males [18]. Li et al. found that it was necessary to avoid modifying the topspin forehand loop technique [14], requiring athletes to hit the ball repeatedly and return the ball with high quality by moving, which ensured that the topspin forehand loop was correct during training so that table tennis athletes could score better in competitions. Lower limbs play a vital role in performance during the topspin forehand loop in racket sports, and the lower limbs have considerable influence on the ball and racket speed as the origin of the kinetic chain [5,19]. He et al. compared the differences between diagonal and straight shots during the forehand loop and found that the straight shot had larger range of motion (ROM) of the ankle plantar flexion external rotation. Compared with straight shots, ROM of diagonal shots of the knee extension significantly increased. In addition, the knee internal rotation of the straight shots significantly increased [20]. The above research showed that investigations into the lower limb biomechanical characteristics during the topspin forehand loop were necessary. These studies suggested that it was necessary to research the biomechanics, especially lower limb characteristics in table tennis [21]. 

The lower limb biomechanics of the topspin forehand loop during table tennis performance have attracted many researchers’ studies. Iino et al. found advanced players showed a significantly larger contribution of lower trunk axial rotation to the racket speed at impact and a significantly larger value of max. Advanced players tended to require less time for racket acceleration than the intermediate players; the ability to accelerate the racket in less time in the topspin forehand against backspin balls may be an important factor that affects the performance level [21]. Qian et al. compared intermediate athletes with superior athletes. There was a statistical difference in completing the forehand loop; the completion of the superior athletes’ was faster. The superior athletes showed a significantly larger external rotation of the knee joint as well as flexion of the hip joint in the backward end. A significantly larger hip internal rotation and extension showed at the forward end. The skills of athletes became more accurate and then improved. Elite table tennis athletes had lower extremities that were better at driving the forehand loop when they compared biomechanics information of lower limbs between different levels of table tennis players during the forehand loop [22,23]. By analyzing the biomechanics of the techniques of table tennis, they not only understand the techniques of the topspin forehand loop but can also analyze the characteristics at different levels. Visual-3D was a commercial software primarily used for motion analysis and biomechanical research. It could evaluate athletes’ performances and movement skills by processing and analyzing motion capture data [24,25,26]. He et al. analyzed the techniques of the topspin forehand loop at different levels with Visual-3D. Elite athletes showed significantly less knee as well as hip flexion in the backward-swing. Moreover, elite athletes had a significantly larger ankle plantarflexion as well as dorsiflexion than medium athletes in the backward-swing and forward-swing phase [20]. 

Previous studies based on Visual-3D, specifically about table tennis, were limited to joint angles, joint moments, and limb center of gravity metrics, but more precision and more metrics were needed to demonstrate. Visual-3D was capable of accepting data from 3D motion capture systems, which could be used to create a skeletal model of the human body for the purpose of conducting motion analysis [27,28]. These data could be imported into the software, and its built-in algorithms and tools could be used for kinematic analysis. With Visual3D, users could create 3D skeletal models, analyze joint movements, calculated biomechanical parameters, and generate reports [25]. To verify the results of Visual-3D, Teoh et al. processed two motion files using Matlab [27,29], generated a virtual hip joint using Visual-3D, and compared the error levels of the motion paths for each reflective point. However, Visual-3D was unable to move the reflective point positions; thus, we calculated the coordinate system for the motion path. Delp et al. used OpenSim [30] to adjust the parameters by scaling the model and optimized the value of h to minimize simulation errors. The mean error at the reflection points in our study was less than 0.02 m, and the maximum error was less than 0.03 m. Our objective was to determine the optimal input parameters to produce a simulation that accurately tracked the experimental data. The biomechanics of lower extremities at different levels during the table tennis forehand loop in OpenSim have not been studied in detail. OpenSim is a free, open-source platform for generating and executing dynamic simulation and analysis, which provides tools for solving inverse kinematics [30,31,32]. OpenSim could match marker trajectories collected in the motion capture system to the virtual makers in the model, enabling an authentic and reliable musculoskeletal model. The program allows the user to manipulate the complex motions and forces involved in musculoskeletal systems [33]. Therefore, it was necessary to study the biomechanics of the topspin forehand loop of table tennis athletes at different levels and adjust training plans according to various personal needs of table tennis athletes in new ways [34].

The hypothesis of this study posited that there would be significant differences in kinematics of the lower limbs during forehand loops between NL1 and NL2. Specifically, NL1 was expected to exhibit greater lower limb angles and range of motion during the topspin forehand loop. The study aimed to determine if lower limb joint angles and range of motion differed between different levels of topspin forehand loops via OpenSim Musculoskeletal Modelling. In this study, OpenSim Musculoskeletal Modelling was used to analyze the joint angles and ROM of lower extremities, and the entire motion time was processed to analyze the different levels. The study’s objective was to provide effective theoretical references for table tennis and practical guidance for coaches in topspin forehand loop training. Moreover, athletes could formulate individualized training plans to enhance their performances based on the study’s findings.

## 2. Materials and Methods

### 2.1. Participants

This study involved 20 table tennis athletes; the sample size was calculated using G*Power 3.1 (Franz Faul, Germany). The estimated effect size for our study was set to 0.7 [35]. The alpha level was set to 0.05, and the power was set to 0.8. Using the ANCOVA test and an effect size of 0.7, it was calculated that a total of 18 participants were required for our study [36], as outlined in Table 1. Ten elite athletes (National Level I Athletes) and ten medium athletes (National Level II Athletes) with average ages of 20 ± 1.20 and 22 ± 1.50 years old and average heights of 174 ± 4.22 and 173 ± 4.59 cm, respectively. The average weights were 68 ± 12.50 and 74 ± 6.25 kg, respectively. All participants were members of the Ningbo University Table Tennis Team, who were right-handed without lower extremity injuries or surgeries at least six months prior to the study. All participants were required to perform the topspin forehand loop with chasse step. The participants were informed of the test procedures and requirements before the study and completed the experimental agreements. The study protocol was approved by the Ethics Committee of Research Academy of Grand Health at Ningbo University (RAGH20210910). 

### 2.2. Experimental Setup 

Prior to the formal experiment, the height and weight values for all participants were recorded with a calibrated weighing scale and stadiometer. The kinematic information was collected by an eight-camera Vicon motion capture system (Oxford Metrics Ltd., Oxford, UK) at a frequency of 200 Hz during the test. There were a total of fifty-two reflective markers (diameter: 14 mm), including left and right shoulders, distal joints of the humerus, radius and ulna, the proximal joints of the second and fifth phalanx, iliac spine, condyle, malleolus, first and fifth metatarsal heads, distal joints of the first and second toe, as well as tracking clusters attached to the elbow, wrist, thigh, shank, and heel. The infrared cameras in the Vicon system captured the reflection or emission of infrared signals from the markers.

To ensure the ability to capture the entire motion process, the camera positioning met the following criteria. Firstly, the camera should cover all key points of the object to be tracked as much as possible and should avoid mutual occlusion. The experimental camera was positioned 2 m from the table tennis table. Secondly, the camera angle should be as perpendicular as possible to the motion trajectory of the tracked object to minimize the effect of visual occlusion and projection distortion. The camera focus points should be set within the subject’s trajectory. Lastly, the height of the camera should be determined according to the height and motion range of the tracked object to ensure that the camera can capture the entire motion trajectory. For this experiment, the height of the experimental camera was set to 1.5 m.

**Figure 1:**
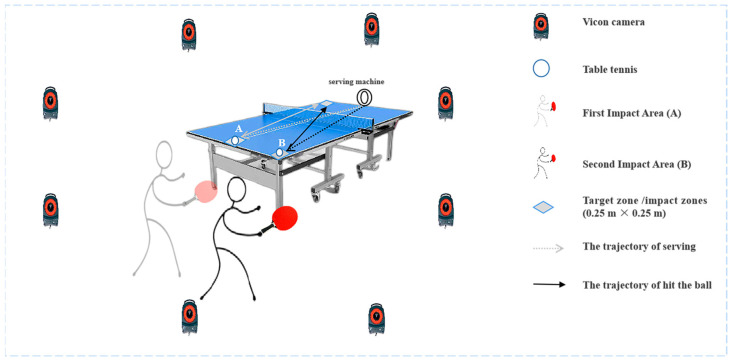
Experimental set up, including the backward phase (BP) and forward phase (FP) during topspin forehand loop.

Before the data collection, all participants were familiar with the experimental environment. They were asked to do a specific 20 min warm-up, which included jogging on a professional treadmill and performing a multi-ball training for 10 min with the topspin forehand loop. As shown in Figure 1, the test was conducted at the Table Tennis Training Center at Ningbo University. The experimental process included one target zone and two impact zones (0.25 m × 0.25 m) tracing upon a professional table tennis table (Rainbow, Double Happiness Sports Company, Shanghai, China). Athletes were asked to hit the ball in the first impact area (A), move quickly to the second impact area (B) with a chasse step, and then participants stroked with maximal power to the target zone. Data was collected only when the forehand topspin loop was performed in the second impact area (B). All test balls were served from a serving machine, which had the same angle, speed, and frequency settings. All participants were informed of the experimental requirements and procedures. During the test, all participants wore the same table tennis match shoes and used the same table tennis racket. Participants were asked to hit balls (D40+, Double Happiness Sports Company, Shanghai, China) with their maximum effort onto the target zone (diagonal shot) just as they would do in a formal match. If the balls were hit out of bounds or into the net, the trials were not considered. Each participant successfully completed at least ten trials, and the kinematics of each trial were recorded synchronously. 

### 2.3. Definition of the Motion Phase

**Figure 2:**
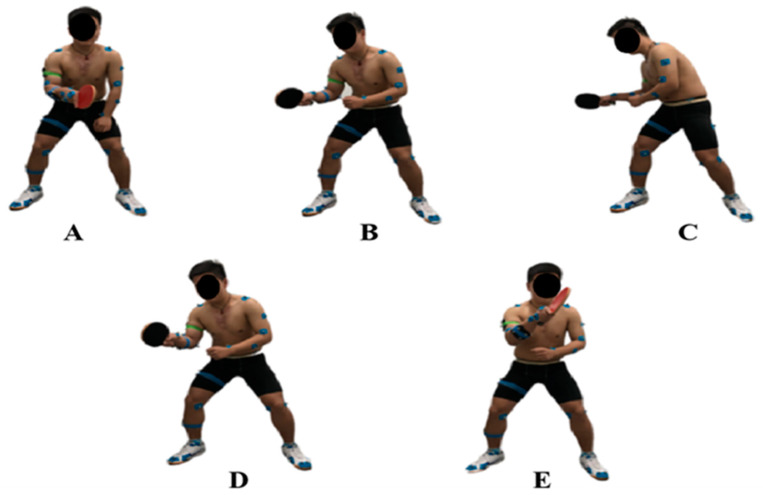
The definition of the motion phase during the test. Position (**A**) was defined as the natural position (NP). (**B**,**C**) shows the backward phase (BP) of the topspin forehand loop. In this study, position (**C**) was defined as the key event of the entire phase of the topspin forehand loop, which is the end of the backward phase (BE). (**D**,**E**) shows the forward phase (FP) during topspin forehand loop, and position E was defined as the end of the forward phase (FE).

The stance interval of the topspin forehand loop in this study is shown in Figure 2. Figure 2A was defined as a natural position (NP). Figure 2B, C shows the backward phase (BP) of the topspin forehand loop. In this study, C was defined as the key event of the entire phase of the topspin forehand loop, which is the end of the backward phase (BE). Figure 2D, E shows the forward phase (FP) during topspin forehand loop, and E was defined as the end of the forward phase (FE).

### 2.4. Data Processing

In this study, the ball was hit to the second impact area (B), and the whole topspin forehand loop motion phase was divided into a forward phase and a backward phase. The swing phase referred to the time interval between two specific events, namely the natural position (NP) and the end of the backward phase (BE, maximum knee flexion) were the backward swing phase, and the backward swing stage included BE and end of the forward phase (FE, maximum hip rotation). Lower extremity joint angles and range of motion (ROM) data were recorded throughout each motion cycle for further processing and analysis. After, the motion was normalized to 101 data points. The extremity performing the strokes was regarded as the dominant side; thus, variables were selected on the dominant side, such as joint angles and range of motion (ROM), for further analysis.

### 2.5. Statistical Analysis

This study used MATLAB scripts to extract joint angles and ROM and to enable differential calculation. The resulting data were imported into MATLAB R2019A (MathWorks, Natick, MA, USA) and processed using scripts that were written for specific data sets. After obtaining with MATLAB, the well-formed files were put into OpenSim. The specific musculoskeletal models were created by scaling general musculoskeletal models (gait2392model) to anthropometric dimensions for each participant. For each topspin forehand loop, the Inverse Kinematics algorithm of OpenSim was used to generate joint angle trajectory. Data were analyzed using SPSS (Version 26.0, Chicago, IL, USA), and the statistical significance level was set to 0.05. Independent t-tests were performed to determine the kinematic differences between the topspin forehand loop of table tennis athletes at different levels in each variable of interest. The analysis included joint angles and range of motion (ROM) of the hip, knee, and ankle joints.

## 3. Results

### 3.1. Motion Time

As shown in Table 2, the motion times of NL1 and NL2 during the topspin forehand loop were 1.45± 0.04 s and 1.65 ± 0.06 s, respectively. There was a significant difference in the entire motion time between NL1 and NL2 (*p* < 0.001). Moreover, the time of NL1 was significantly shorter than NL2 in the FP (*p* = 0.028), but there was no significant difference in BP.

### 3.2. Joint Angles

The joint angles at BE as well as FE between NL1 and NL2 of the hip, knee, and ankle were statistically analyzed, with the results shown in Table 3. In the sagittal plane, compared with NL2, NL1 had a significantly greater hip flexion (*p* = 0.001) and knee flexion (*p* = 0.007) at BE. Moreover, NL1 had a significantly greater hip extension (*p* < 0.001), knee flexion (*p* < 0.001), and ankle flexion (*p* = 0.008) at FE. In the frontal plane, the hip adduction and abduction in NL1 were all less than NL2. In the transverse plane, the hip internal and external rotations of NL1 were greater than NL2. 

**Figure 3:**
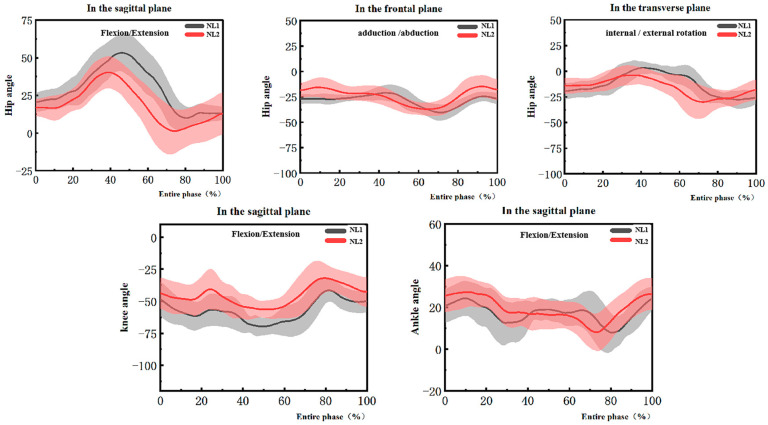
The lower extremity joint angles during the entire phase of the topspin forehand loop between NL1 and NL2.

Figure 3 shows the kinematic differences for the EP of the topspin forehand loop between NL1 and NL2. NL1 shows a longer time to reach the key event of the entire phase and less time to complete the FP during the topspin forehand loop. In the sagittal plane, NL1 had a greater hip flexion and NL2 had greater knee flexion at EP. NL1 displayed less ankle extension at BP and greater ankle flexion at BP. In the frontal plane, NL1 had greater abduction at FP and less hip adduction at EP. In the transverse plane, the hip internal rotation of NL1 was less than NL2 at BP and greater external rotation at FP.

### 3.3. Range of Motion

**Figure 4:**
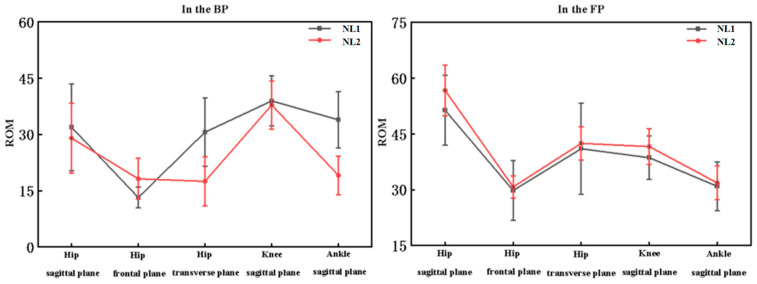
The lower extremity range of motions during topspin forehand loop between NL1 and NL2.

The ROM for NL1 and NL2 during the topspin forehand loop are shown in Table 4 and Figure 4. The lower extremity ROMs of NL1 and NL2 were significantly different at different levels. In the sagittal plane, NL1 significantly had greater flexion and extension at the hip (*p* = 0.019) and ankle (*p* < 0.001) but with no significant difference in knee flexion and extension. In the frontal plane, the ROM of NL1 in the hip joint had significantly less adduction and abduction than NL2 (*p* < 0.001). In the transverse plane, compared with NL2, NL1 had significantly less internal and external rotation (*p* < 0.001) in the hip joint.

The ROM of NL1 and NL2 during the BP and FP are shown in Table 5. The lower limb ROM values for NL1 and NL2 were significantly different in the BP and FP. In the sagittal plane, NL1 displayed less hip extension (*p* = 0.042) and knee extension (*p* = 0.042) at FP and less ankle flexion (*p* = 0.015) at BP. In the frontal plane, NL1 displayed significantly greater hip adduction (*p* = 0.003) at BP. In the transverse plane, NL1 displayed significantly less hip internal rotation at BP.

## 4. Discussion

The purpose of this study was to analyze lower extremity kinematics of table tennis athletes during the topspin forehand loop between NL1 and NL2 via OpenSim Musculoskeletal Modelling. The main findings of this study were as follows: NL1 and NL2 differed significantly in terms of time spent during the entire phase. The time of NL1 was notably shorter than that of NL2 in both the FP and EP. Additionally, there were significant differences in joint angles and range of motion (ROM) during the BP and FP between medium and elite table tennis athletes.

In terms of time, NL1 was shorter than that of NL2 in the forward and backward phase. However, there was a significant difference between NL1 and NL2 in the forward and the entire phases [1,37], indicating that the entire phase of NL1 was shorter, which may prove that NL1 could complete a more effective topspin forehand loop in the limited time than NL2. NL1 takes less time in the FP phase; thus, it could suggest that NL1 had a stronger ability to accelerate the racket quickly. This was consistent with previous research. Qian et al. reported that compared with intermediate athletes, the superior athletes showed less time for the table tennis forehand loop [5]. Table tennis athletes have limited time to hit the ball; thus, it is beneficial for them to improve their swing speed in a short time [38]. Advanced athletes have better body control [39]. This would indicate that elite table tennis athletes adjust the relative and absolute speed and time of racket, which require having better performance in table tennis training or competitions [37]. Therefore, medium table tennis athletes need to decrease the forward swing time to improve their racket speed and enhance their topspin forehand loop ability. 

In the entire phase, the hip flexion and rotation of NL1 were remarkably greater than that of NL2. One of the most critical factors in racket sports was rotation of the trunk axis. In the process of movements, hip movement was an indispensable core. The involvement of the trunk, especially rotation of the hip joint, greatly affects the swing, which is also a criterion to judge the levels of the athletes [40]. Elite athletes have better trunk ability. The rotation of the hip joint increases the swinging motion of the forehand and the rotation of the hip joint increases the initial speed of the arm, thus increasing the speed of the stroke. According to the formula: linear velocity (V) = angular velocity (ω) x radius (r); the ball was hit by the upper arm without the acceleration of the hip joint. The radius of rotation of the stroke is the line from the center of the shoulder joint to the point where the racket touches the ball. The larger the radius, the greater the linear speed, given a certain angular speed [38,41]. Therefore, hip movement plays an essential role of energy generation and transmission in table tennis [42]. Additionally, the core consists of the lumbar pelvis–hip complex and the surrounding muscle tissue [8]. In the training of the forehand loop, it is important to strengthen the training of the core muscle group and improve the speed of ball [43]. Therefore, elite athletes have greater core and hip muscle strength. At the same time, these factors are conducive for high-level athletes to improve performance and for the development of professional careers. In training, athletes could effectively use external resistance (up and down slopes, running steps) for short distance all-out sprint running, double swing jump rope, barbell high flip, and glute bridge, and other training methods could exercise the gluteus maximus, biceps femoris, and quadriceps fast contraction ability [44,45], and improve the ability to turn the hip when performing the topspin forehand loop.

Moreover, NL1 had significantly greater knee flexion and extension. The stability of the knee joint under rapidly changing loads was important, which affected the dynamic stability of the knee joint [46]. Kasai et al. found that elite athletes tended to use their entire body, rotate their upper body, and use their knees more effectively during the topspin forehand loop [47]. Moreover, previous studies pointed out that advanced table tennis athletes took a higher risk of knee injury [40,48]; thus, table tennis athletes of higher levels would have higher threats to knee injury during the topspin forehand loop. Table tennis athletes should pay attention to protecting the knee joint, improving the strength of the knee joint, enhancing the stability of the knee joint, and reducing the possibility of knee injury. Furthermore, compared with the ROM of the entire motion phase, the range of motion of NL1’s ankle joint was significantly larger than NL2. A strong ankle joint helps athletes to support the weight and keep the body stable for better stroke performance [49,50]. Kondric et al. provided epidemiological data on injury localization among top Slovenian racket sports players (table tennis, tennis, and badminton), finding ankles as the site of injury among elite table tennis players (approximately 13%) [51]. Zhang et al. found NL1 showed significantly larger joint motion flexibility and strength of ankle [52]. This could infer that the ankle joint of NL1 had better topspin forehand loop performance, which may make elite athletes have higher potential risks of ankle sprains [42,50]. Therefore, table tennis athletes need to pay attention to increasing the strength of their knee and ankle muscles and to avoiding sports injuries as well as moving up to higher levels.

However, there were some limitations in this study. Firstly, this study only compared the biomechanical differences of dominant lower limbs, but non-dominant limbs also played a crucial role. Secondly, this study only captured the kinematics data for the topspin forehand loop; other kinetics data would also need to be collected. In future studies, the relationships between the upper and lower extremity could be considered.

## 5. Conclusions

This study systematically analyzed the lower extremity kinematics of table tennis athletes performing the topspin forehand loop using OpenSim Musculoskeletal Modelling. The study introduced the movement of lower extremity joints in table tennis athletes at different levels. The results showed that: (1) There was a significant difference between elite athletes and medium athletes in the forward and entire phases. Elite athletes were faster during the topspin forehand loop, and NL2 needed to improve stroke speed. (2) Elite athletes had full hip and knee movement in the EP and better lower extremity drive ability during the topspin forehand loop than medium athletes. Table tennis athletes need to improve their core and hip muscle trunk drive abilities, particularly the core and hip muscles, through exercises to achieve stable control of the center of gravity, improve the speed of rapid braking and starting, have efficient transmission of strength, and prevent injury. (3) The power of elite athletes was more fully concentrated on the topspin forehand loop, suggesting that coaches developing a physical training plan could choose training methods to develop the flexibility and strength training of the athlete’s knee and ankle joints. Table tennis was commonly regarded as a reactive sport, owing to the rapid ball speed and the close proximity of players. Indeed, athletes were afforded only a brief moment to execute a return (less than one second). During the limited time, the table tennis player must make a decision as to how to stroke and then adjust to the appropriate ready position. As the findings of previous research, elite table tennis athletes exhibit greater range of motion within a shorter time compared to their counterparts [21]. This suggests that elite players in table tennis possess the capacity for faster movement, resulting in correspondingly rapid ball speed in comparison to those with lower skill levels [53].

## Figures and Tables

**Table 1:** The participants’ characteristics information (Mean ± SD).

Population	Level	Age (Year)	Height (cm)	Weight (kg)
10	NL1	20 ± 1.20	174 ± 4.22	68 ± 12.50
10	NL2	22 ± 1.50	173 ± 4.59	74 ± 6.25

Note: NL1, elite athletes; NL2, medium athletes.

**Table 2:** Comparison motion times between NL1 and NL2 (unit: seconds).

Phase	NL1	NL2	*p*-Value
	Mean ± SD	Mean ± SD	
BP	0.45 ± 0.06	0.55 ± 0.06	0.593
FP	0.95 ± 0.06	1.15 ± 0.08	0.028 *
EP	1.45 ± 0.04	1.65 ± 0.06	0.000 *

Note: * Significant differences between NL1 and NL2 (*p* < 0.05). NL1, elite athletes; NL2, medium athletes. BP, backward phase; FP, forward phase; EP, entire phase.

**Table 3:** Comparison of joint angles in key events between NL1 and NL2 (unit: degrees).

Index		NL1		NL2		*p*-Value
		Phase	Mean ± SD	Phase	Mean ± SD	
Hip	In the sagittal plane	BE	62.94 ± 2.03 *	BE	47.94 ± 7.64 *	0.001 *
		FE	3.92 ± 3.41 *	FE	−5.34 ± 10.96 *	0.001 *
	In the frontal plane	BE	−14.78 ± 2.04 *	BE	−7.00 ± 6.65 *	0.001 *
		FE	−45.90 ± 2.35 *	FE	−40.74 ± 3.91 *	0.002 *
	In the transverse plane	BE	8.35 ± 3.86 *	BE	3.43 ± 4.94 *	0.002 *
		FE	−34.87 ± 2.73 *	FE	−39.87 ± 10.53 *	0.001 *
Knee	In the sagittal plane	BE	−30.30 ± 5.34 *	BE	−20.83 ± 7.78 *	0.007 *
		FE	−75.59 ± 2.38 *	FE	−64.88 ± 4.68 *	0.001 *
Ankle	In the sagittal plane	BE	34.08 ± 3.09	BE	32.91 ± 3.97	0.061
		FE	−5.60 ± 3.85 *	FE	−1.52 ± 4.79 *	0.008 *

Note: * Significant differences between NL1 and NL2 (*p* < 0.05). NL1, elite athletes; NL2, medium athletes. BE, the end of the backward phase; FE, the end of the forward phase.

**Table 4:** Comparison of means ± standard deviations for ROM between NL1 and NL2 at the entire phase during topspin forehand loop (unit: degrees).

Index		NL1	NL2	*p*-Value
		Mean ± SD	Mean ± SD	
Hip	In the sagittal plane	59.11 ± 4.14 *	53.28 ± 7.36 *	0.019 *
	In the frontal plane	30.98 ± 2.94 *	33.74 ± 5.95 *	0.000 *
	In the transverse plane	43.33 ± 4.44	43.30 ± 12.00	0.000 *
Knee	In the sagittal plane	45.19 ± 6.00	44.05 ± 7.14	0.626
Ankle	In the sagittal plane	39.35 ± 3.99 *	34.43 ± 5.27 *	0.000 *

Note: * Significant differences between NL1 and NL2 (*p* < 0.05). NL1, elite athletes; NL2, medium athletes. BE, the end of the backward phase; FE, the end of the forward phase.

**Table 5:** Comparison of means ± standard deviations for ROM in the BP and FP between NL1 and NL2 during topspin forehand loop (unit: degrees).

Index		NL1		NL2		*p*-Value
		Phase	Mean ± SD	Phase	Mean ± SD	
Hip	In the sagittal plane	BP	29.02 ± 9.27	BP	31.91 ± 11.57	0.171
		FP	51.36 ± 9.35 *	FP	56.71 ± 6.80 *	0.042 *
	In the frontal plane	BP	18.17 ± 5.46 *	BP	13.24 ± 2.77 *	0.003 *
		FP	29.80 ± 8.05	FP	30.77 ± 2.96	0.590
	In the transverse plane	BP	17.51 ± 6.57 *	BP	30.62 ± 9.12 *	0.024 *
		FP	41.02 ± 12.20	FP	42.47 ± 4.47	0.841
Knee	In the sagittal plane	BP	37.83 ± 6.39	BP	38.94 ± 6.64	0.153
		FP	38.62 ± 5.87 *	FP	41.62 ± 4.80 *	0.042 *
Ankle	In the sagittal plane	BP	19.10 ± 5.15 *	BP	33.90 ± 7.46 *	0.015 *
		FP	30.92 ± 6.56	FP	31.85 ± 4.54	0.581

Note: * Significant differences between NL1 and NL2 (*p* < 0.05). NL1, elite athletes; NL2, medium athletes. BP, backward phase; FP, forward phase.

## Data Availability

The data that support the findings of this study are available on reasonable request from the corresponding authors. The data are not publicly available due to privacy or ethical restrictions.
